# Alveolar Macrophages Are Essential for Protection from Respiratory Failure and Associated Morbidity following Influenza Virus Infection

**DOI:** 10.1371/journal.ppat.1004053

**Published:** 2014-04-03

**Authors:** Christoph Schneider, Samuel P. Nobs, Alex K. Heer, Michael Kurrer, Glynis Klinke, Nico van Rooijen, Johannes Vogel, Manfred Kopf

**Affiliations:** 1 Molecular Biomedicine, Institute of Molecular Health Sciences, Department of Biology, ETH Zurich, Zurich, Switzerland; 2 Pathology Institute, Zurich, Switzerland; 3 Division of Clinical Chemistry and Biochemistry, University Children's Hospital Zurich, Zurich, Switzerland; 4 Department of Molecular Cell Biology, Free University Medical Center, Amsterdam, The Netherlands; 5 Institute of Veterinary Physiology, University of Zurich, Zurich, Switzerland; Johns Hopkins University - Bloomberg School of Public Health, United States of America

## Abstract

Alveolar macrophages (AM) are critical for defense against bacterial and fungal infections. However, a definitive role of AM in viral infections remains unclear. We here report that AM play a key role in survival to influenza and vaccinia virus infection by maintaining lung function and thereby protecting from asphyxiation. Absence of AM in GM-CSF-deficient (*Csf2* ^−/−^) mice or selective AM depletion in wild-type mice resulted in impaired gas exchange and fatal hypoxia associated with severe morbidity to influenza virus infection, while viral clearance was affected moderately. Virus-induced morbidity was far more severe in *Csf2* ^−/−^ mice lacking AM, as compared to *Batf3*-deficient mice lacking CD8α^+^ and CD103^+^ DCs. *Csf2* ^−/−^ mice showed intact anti-viral CD8^+^ T cell responses despite slightly impaired CD103^+^ DC development. Importantly, selective reconstitution of AM development in *Csf2rb* ^−/−^ mice by neonatal transfer of wild-type AM progenitors prevented severe morbidity and mortality, demonstrating that absence of AM alone is responsible for disease severity in mice lacking GM-CSF or its receptor. In addition, CD11c-Cre/*Pparg* ^fl/fl^ mice with a defect in AM but normal adaptive immunity showed increased morbidity and lung failure to influenza virus. Taken together, our results suggest a superior role of AM compared to CD103^+^ DCs in protection from acute influenza and vaccinia virus infection-induced morbidity and mortality.

## Author Summary

Acute respiratory viral infections can cause severe morbidity and pneumonia in infected individuals. Alveolar macrophages and various subsets of dendritic cells have been implicated in innate immunity and induction of anti-viral T cell responses that contribute to host defense against influenza virus infection. However, their relative importance in protection from pathology and disease severity has never been compared side by side. In this report, we demonstrate that mice lacking alveolar macrophages succumb to infection with low dose influenza virus and vaccinia virus infection due to respiratory failure. In contrast, mice lacking lymphoid CD8α^+^ and lung CD103^+^ DCs survived and showed little if any differences in disease severity compared to infected wild-type mice. These results indicate that therapies supporting AM and lung function may be beneficial during severe respiratory viral infection.

## Introduction

Alveolar macrophages (AM) are lung-resident macrophages important for the maintenance of surfactant homeostasis in the alveolar space [1]. Their importance for lung physiology becomes evident in a rare human syndrome termed “pulmonary alveolar proteinosis” (PAP), which is characterized by the accumulation of surfactant material and a varying degree of respiratory insufficiency [2]. PAP patients have a higher risk for pulmonary infections with opportunistic pathogens [3]. PAP typically occurs in patients that spontaneously develop GM-CSF autoantibodies [4] or carrying mutations in the GM-CSF receptor α chain [5] associated with impaired function and/or reduced numbers of AM. Similarly, mice lacking GM-CSF (*Csf2* ^−/−^) or the receptor β chain (*Csf2rb* ^−/−^) develop PAP [6], [7], [8], [9] and display increased susceptibility to a range of bacterial and fungal infections, which is associated with impaired innate functions of AM [10], [11], [12], [13], [14]. High phagocytic activity and expression of pattern recognition receptors provides them with the capacity to respond to bacterial and fungal pathogens. However, AM were also described to have anti-inflammatory properties based on direct inhibition of the antigen-presenting function of lung DCs [15] and production of immunosuppressive mediators such as IL-10 and nitric oxide [16]. In addition, AM were proposed to sequester pulmonary antigen and thereby interfere with efficient priming of immune responses by DCs [17]. In contrast to the well-described functions of AM in bacterial and fungal infections, their precise role during viral infections is poorly understood. AM have the capacity to endocytose adenovirus particles, which was impaired in cells isolated from *Csf2*-deficient mice [10]. Furthermore, AM are potent producers of type I IFNs upon pulmonary virus infection [18], [19]. A beneficial role of AM in influenza infection has been proposed based on AM depletion experiments [20], [21], [22] and treatment of mice with GM-CSF that increased numbers of AM [23], [24]. However, besides the described role of GM-CSF for AM function under steady-state conditions, GM-CSF also influences DCs as demonstrated by its positive effects on the homeostasis of CD103^+^ DCs [25] or the upregulation of CD103 on CD11b^−^ DCs [26]. Indeed, a recent report proposed that GM-CSF protects from lethal influenza virus infection by enhancing CD103^+^ DC mediated anti-viral T cell responses [27].

In this study, we revisited these controversies by comparing DC subpopulations and anti-viral CD8^+^ T cell responses in the presence or absence of AM with the outcome of respiratory viral infection in *Csf2* ^−/−^, *Csfrb2* ^−/−^, *Batf3* ^−/−^, CD11c-Cre/*Pparg* ^fl/fl^ (with a defect in AM) and wild-type mice. *Csf2*-deficient mice developed severe morbidity and hypersuceptibility to influenza virus and vaccinia virus infection due to the complete absence of AM, although CD103^+^ DC mediated anti-viral T cell responses were unaffected. In contrast, virus-induced morbidity was moderately affected in *Batf3*-deficient mice, which mounted impaired anti-viral T cell responses due to the absence of CD8α^+^ DCs and lung CD103^+^ DCs. Selective depletion of AM using clodronate liposomes prior influenza virus infection resulted in morbidity comparable to *Csf2*-deficient mice. Moreover, mice with functionally impaired AM due to conditional deletion of *Pparg* showed increased disease severity and respiratory failure despite normal T cell responses. In contrast, selective postnatal AM reconstitution in *Csfrb2* ^−/−^mice prevented pulmonary pathology and morbidity following influenza virus infection. Overall, our study provides evidence for a vital function of AM for the maintenance of lung function upon pulmonary viral infections.

## Results

### Alveolar macrophages fail to develop in the absence of GM-CSF production by non-hematopoietic cells

**Figure 1:**
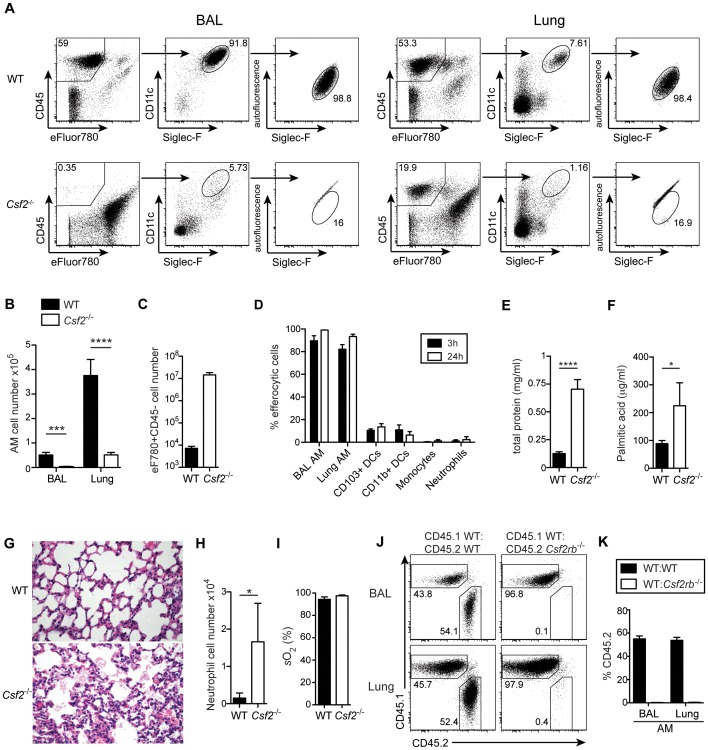
Cell-intrinsic requirement of GM-CSF for the development of alveolar macrophages. (A) Identification of live (eFluor780^−^) AM (CD45^+^CD11c^+^Siglec-F^+^ autofluorescent^high^) in BAL and lung of WT and *Csf2* ^−/−^ mice. (B) Absolute cell numbers of AM. (C) Quantification of dead CD45^−^eFluor780^+^ cells in the BAL. (D) Efferocytosis of i.t. instilled apoptotic thymocytes by indicated populations of myeloid cells. Values shown depict percentages of efferocytic cells. (E and F) Concentrations of total protein (E) and palmitic acid (F) were measured in the BAL of WT and *Csf2* ^−/−^ mice. (G) Panels show H&E-stained histological lung sections of WT and *Csf2* ^−/−^ mice. (H) Absolute cell numbers of neutrophils (CD11b^+^CD11c^−^Gr-1^+^) in the BAL. (I) Arterial oxygen saturation in WT and *Csf2* ^−/−^ mice. (J and K) Mixed BM chimeras (1∶1 mixture of CD45.1^+^WT∶CD45.2^+^ *Csf2rb* ^−/−^ or CD45.1^+^WT∶CD45.2^+^WT) were analyzed for the contribution of CD45.1 and CD45.2 BM to the development of AM. (J) Dot plots show the percentage of CD45.1^+^WT and CD45.2^+^ *Csf2rb* ^−/−^ (or control CD45.2^+^WT) cells among CD11c^+^Siglec-F^+^ AM in the BAL and lung. (K) Bar graphs display the frequency of CD45.2^+^WT and CD45.2^+^ *Csf2rb* ^−/−^ AM as gated in (J). The mean ± SD is shown.

Mice lacking GM-CSF (*Csf2* ^−/−^) or the β subunit of its receptor (*Csf2rb^−/−^*) have been reported to develop alveolar proteinosis due to a defect in terminal maturation and surfactant catabolism of AM [6], [8]. However, AM characterized as CD45^+^CD11c^+^Siglec-F^+^ cells with high autofluorescence were completely absent in lung and BAL of adult *Csf2* ^−/−^ mice (Figure 1A, B and Figure S1). Staining with a viability dye showed a striking enrichment of dead non-hematopoietic (CD45^−^) cells, presumably consisting mainly of lung epithelial cells (Figure 1C), indicating that the removal of dead cells (efferocytosis) in the respiratory tract is impaired in the absence of AM. Indeed, following tracheal instillation of labeled apoptotic thymocytes, we found that AM are by far the most potent cell type compared to DCs and neutrophils in removal of apoptotic cells from the bronchoalveolar space (Figure 1D and Figure S1). The few remaining cells that showed up in the CD11c^+^Siglec-F^+^ population of *Csf2* ^−/−^ mice were contaminating dead cells with high autofluorescence as demonstrated by comparing the analysis with and without prior exclusion of dead cells (Figure S1). Absence of AM resulted in the accumulation of lipoproteinaceous and eosinophilic material in the BAL and lung of *Csf2* ^−/−^ mice (Figure 1E–G). Moreover, while BAL of WT mice consisted almost entirely (>90%) of AM, BAL of *Csf2* ^−/−^ mice contained a substantial proportion of neutrophils (Figure 1H). This indicates low-grade inflammation possibly induced by the accumulation of dead cells (Figure 1C). Nonetheless, arterial oxygen saturation in *Csf2* ^−/−^ and WT mice was comparable indicating unimpaired gas exchange in the lung in the absence of AM under steady state (Figure 1I).

To determine whether AM require cell intrinsic GM-CSFR signaling for their development, we generated mixed bone marrow (BM) chimeras by adoptive transfer of a 1∶1 mixture of BM cells from *Csf2rb* ^−/−^ (CD45.2^+^) and WT (CD45.1^+^) mice (as well as CD45.2^+^WT and CD45.1^+^WT BM as controls). The reconstituted AM in the BAL and lung of *Csf2rb* ^−/−^:WT chimeras were exclusively of WT donor origin and prevented development of alveolar proteinosis. (Figure 1 J, K and data not shown). In agreement with a recent report, these data show a requirement of GM-CSF signaling for the reconstitution of AM post-irradiation [28].

GM-CSF can be expressed by a variety of different cell types including T cells, macrophages, mast cells and non-hematopoietic cells [29]. To assess the source of GM-CSF for the development of alveolar macrophages, we generated 4-way *Csf2* ^−/−^ and WT crisscross BM chimeras. Irradiated WT recipients that received BM from either *Csf2* ^−/−^ or WT mice did not develop any pulmonary alveolar proteinosis during 16 weeks post-reconstitution. In contrast, *Csf2* ^−/−^ recipients reconstituted with WT or *Csf2* ^−/−^ BM showed a massive and comparable proteinosis (Figure S2). These results identify radio-resistant non-hematopoietic cells such as epithelial cells as the primary source of GM-CSF in the lung, which is absolutely essential for development of alveolar macrophages.

**Figure 2:**
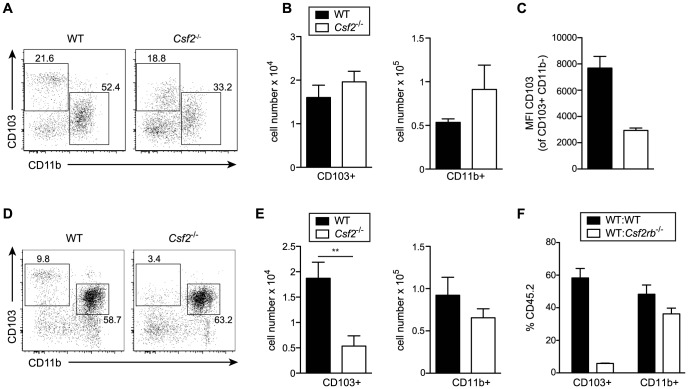
GM-CSF promotes development of CD103^+^ DCs and expression of CD103 in young and adult mice, respectively. Analysis of lung DC subsets in (A–C) 6–8 week-old and (D–E) 6-day-old mice. (A, D) Gated cells in dot plots show CD103^+^ and CD11b^+^ DCs with values indicating percentages among CD11c^+^Siglec-F^−^ lung DCs. Cells were pre-gated on CD45^+^eFluor780^−^ viable cells. (B,E) Bar graphs depict total number of CD103^+^ and CD11b^+^ DCs in the lung. (C) Mean fluorescence intensity (MFI) of CD103 expression on CD103^+^CD11b^−^ DCs. Values shown represent the mean ± SD (n = 3–4). (F) Analysis of lung DC subsets in mixed BM chimeras generated by transfer of 1∶1 mixture of CD45.1^+^WT∶CD45.2^+^ *Csf2rb* ^−/−^ or control CD45.1^+^WT∶CD45.2^+^WT into lethally irradiated recipients. Bar graphs show the percentage of CD45.2^+^WT and CD45.2^+^ *Csf2rb* ^−/−^ cells among CD103^+^ and CD11b^+^ DCs in the lung. The mean ± SD is shown (n = 3).

Development of lung CD103^+^ DCs has been reported to depend on GM-CSF [25]. However, comparing adult WT and *Csf2* ^−/−^ mice, we found similar numbers of pulmonary CD103^+^ DCs, although CD103 surface expression on CD11b^−^CD11c^+^ DCs was strongly impaired in *Csf2* ^−/−^ mice (Figure 2A–C) consistent with a previous study using *Csf2rb* ^−/−^ mice [26]. Interestingly, CD103^+^ DCs were strongly reduced in 6-day-old *Csf2* ^−/−^ pups (Figure 2D, E). In addition, injection of BM from *Csf2rb* ^−/−^ donors to irradiated WT recipients failed to reconstitute pulmonary CD103^+^ DCs (Figure 2F) [25], while other myeloid cell populations in the lung and peripheral blood were comparably well reconstituted from *Csf2rb* ^−/−^ and WT bone marrow cells (Figure S2). These data suggest that GM-CSF plays an important role in development of CD103^+^ DCs, although a population of CD103^low^CD11b^−^ DCs can substitute typical CD103^+^ DCs in the absence of GM-CSF possibly under inflammatory conditions.

### *Csf2* ^−/−^ mice have a reduced resistance to influenza infection despite intact anti-viral T and B cell responses

**Figure 3:**
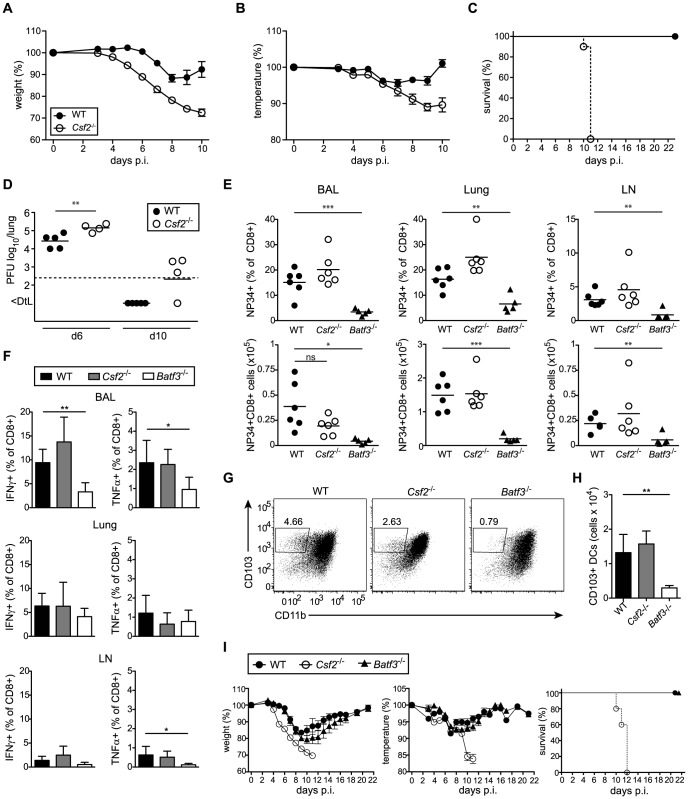
*Csf2* ^−/−^ mice succumb to influenza virus infection despite intact antiviral T and B cell responses. (A–I) Indicated groups of mice were infected i.t. with PR8 influenza virus (50 pfu unless otherwise specified). Loss of body weight (A) and temperature (B) was monitored. (C) Survival after infection with 100 pfu PR8. Values indicate mean ± SEM of 8–10 mice per group. (D) Virus titers in the lung at days 6 and 10 p.i. were measured by plaque-assay. (E–H) Comparison of immune responses in *Csf2* ^−/−^, *Batf3* ^−/−^ and WT mice at day 10 post infection. (E) Percentages (upper panel) and total numbers (lower panel) of influenza NP34-specific CD8^+^ T cells in the BAL, lung and lung-draining LN. (F) Virus-specific IFNγ and TNFα production in CD8^+^ T cells of the BAL, lung and LN was analyzed by restimulation with virus-loaded BMDCs. (G and H) CD103^+^ DCs in the lung at d10 post-infection were gated on eF780^−^CD45^+^CD11c^+^MHCII^+^B220^−^Siglec-F^−^ cells. Dot plots show the frequencies of CD103^+^ DCs of individual mice representative for the group (G) and bar graphs display total numbers (H). Values indicate mean ± SD of 5–6 mice per group. (I) WT, *Csf2* ^−/−^ and *Batf3* ^−/−^ mice were monitored for body weight, body temperature and the survival during the course of infection. Data show mean ± SEM.

To determine whether absence of AM and/or impaired CD103^+^ DC compartment affect resistance of *Csf2* ^−/−^ mice to respiratory viral infection, we infected WT and *Csf2* ^−/−^ mice with a sublethal dose of influenza virus PR8. Compared to WT mice, *Csf2* ^−/−^ animals showed an earlier onset of disease and a much more pronounced loss of body weight and body temperature (Figure 3A, B). Strikingly, whereas all WT mice recovered from infection, moribund *Csf2* ^−/−^ mice had to be euthanized between day 10 and 12 post-infection (Figure 3C). Moreover, *Csf2* ^−/−^ mice showed an increased lung virus titer at the peak of viral load (i.e. day 6) and a slight delay in viral clearance (Figure 3D), which were nevertheless to small to explain the moribund state of the *Csf2* ^−/−^ mice. Importantly, the fatal outcome of infection resulted from the absence of GM-CSF production by non-hematopoietic cells as demonstrated by a higher susceptibility of WT→*Csf2* ^−/−^ compared to *Csf2* ^−/−^→WT BM chimeras (Figure S3).

Lung CD103^+^ DCs have been reported to induce CD8^+^ T cell responses by cross-presentation of influenza virus [19], [30]. Notably, *Csf2* ^−/−^ mice mounted intact anti-viral CD8^+^ T cell effector responses in BAL, lung, and draining LN (Figure 3E, F) despite a 2-fold reduction in lung CD103^+^ DC frequencies and decreased CD103 surface levels during influenza virus infection (Figure 3G). Nonetheless, total numbers of CD103^+^ DCs were similar in *Csf2* ^−/−^ compared to wild-type mice (Figure 3H). In contrast, influenza virus infected *Batf3*-deficient mice were almost entirely devoid of lung CD103^+^ DCs (Figure 3G, H) and showed a strong reduction in virus-specific CD8^+^ T cells and anti-viral effector cytokine (i.e. TNFα and IFNγ) production [19]. Regardless, morbidity and disease severity remained almost completely unaffected in *Batf3*-deficient compared to wild-type mice, while *Csf2* ^−/−^ mice succumbed to infection (Figure 3I). Notably, *Batf3* ^−/−^ mice were able to efficiently clear the virus despite impaired anti-viral CD8^+^ T cell responses (Figure S4).

To further characterize the adaptive response and rule out a possible defect in anti-viral antibody responses in the absence of GM-CSF, we assessed anti-viral B cell responses by measurement of influenza HA-specific antibodies. *Csf2* ^−/−^ mice showed increased levels of virus-specific IgA and IgG2a antibodies in BAL and serum compared to wild-type mice (Figure S4). Furthermore, we found similar NK cell recruitment and increased activation in *Csf2* ^−/−^ compared to wild-type mice (Figure S4).

Together these results demonstrate that CD103^+^ DC mediated anti-viral CD8^+^ T cell responses or B cell responses are intact and therefore not the underlying reason for the disease severity in *Csf2* ^−/−^ mice.

### AM are crucial for the maintenance of respiratory function during influenza virus infection

**Figure 4:**
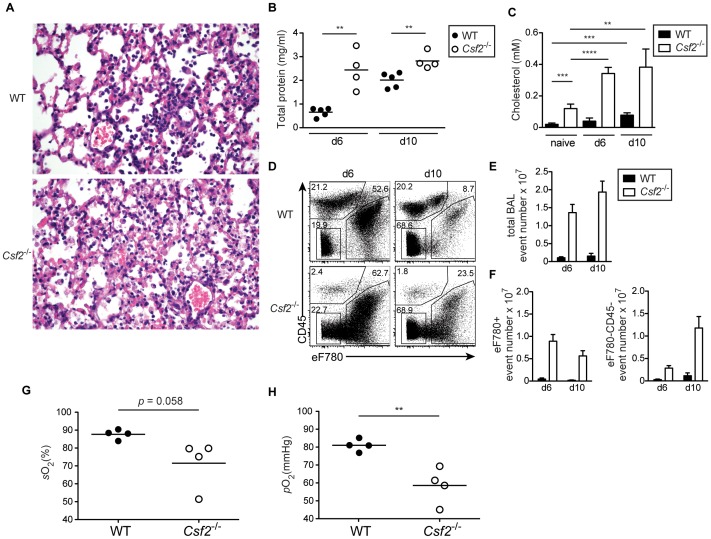
Defective gas exchange and respiratory failure following influenza virus infection in *Csf2* ^−/−^ mice lacking AM. WT and *Csf2* ^−/−^ mice were infected i.t. with 50 pfu PR8 influenza virus. (A) Panels show H&E-stained histological lung sections of day 10-infected WT and *Csf2* ^−/−^ mice. The concentration of total protein (B) and cholesterol (C) in the BAL was determined at the indicated time points after infection. (D) Percentages of dead cells (eFluor780^+^) and debris (eFluor780^−^CD45^−^) in the BAL were determined by flow cytometry at d6 and d10. Bar graphs show the total BAL event number (E) and numbers of eFluor780^+^ or eFluor780^−^CD45^−^ events (F). Shown is the mean ± SD of 4–5 mice per group. Arterial blood oxygen saturation (G) and O_2_ partial pressure (H) was measured in infected animals at day 9. Symbols represent values of individual mice and the mean is shown.

As shown above, absence of AM in mice lacking GM-CSF results in accumulation of surfactant material and dead cells under homeostatic conditions. Interestingly, influenza virus infection strikingly aggravated pulmonary alveolar proteinosis in *Csf2* ^−/−^ mice indicated by an obstruction of alveoli with aggregates of eosinophilic material (Figure 4A), increased total protein concentration (Figure 4B) and accumulation of surfactant material in the BAL fluid at days 6 and 10 p.i. (Figure 4C). Moreover, the BAL of *Csf2* ^−/−^ mice was highly enriched in dead cells and cellular debris indicating impaired clearance of apoptotic cells (Figure 4D–F). Accordingly, respiratory function as measured by arterial oxygen levels was critically impaired at the time when *Csf2* ^−/−^ mice succumb to infection (Figure 4 G, H). These results suggest that AM prevent influenza induced morbidity by maintenance of lung function through removal of dead cells and surfactant material.

### Selective restoration of AM development in *Csf2rb* ^−/−^ mice prevents severe morbidity and mortality following influenza virus infection

**Figure 5:**
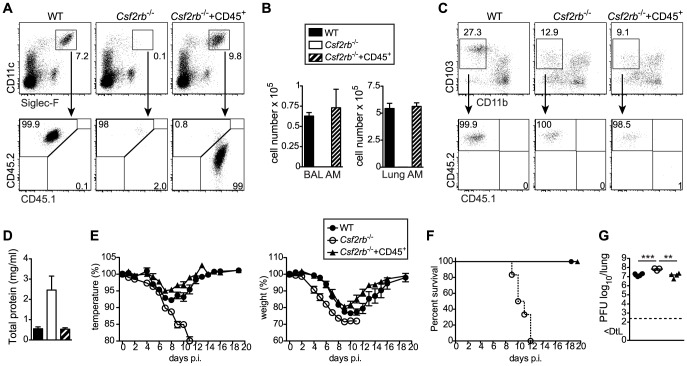
Selective restoration of AM development in *Csf2rb* ^−/−^ mice prevents severe morbidity and mortality following influenza virus infection. Whole CD45^+^ cells containing progenitors of AM were sorted from lungs of CD45.1^+^ E18.5 embryos by flow cytometry and transferred intranasally into neonatal CD45.2^+^ *Csf2rb* ^−/−^ mice. (A) Six weeks after transfer, recipient mice were analyzed for the presence of donor-derived AM. (B) Bar graphs display the total AM cell number in the BAL and lung. (C) Dot plots depict the expression of CD103 and CD11b on CD11c^+^MHCII^+^Siglec-F^−^ lung DCs and frequencies of CD45.1^+^ (donor-derived) and CD45.2^+^ (recipient-derived) cells among CD103^+^ DCs are shown. (D–G) Eight weeks after transfer, recipient mice were infected with 50 pfu PR8 influenza virus. (D) Total protein concentration in the BAL at d5 after infection. (E) Loss of body weight and temperature and (F) survival was monitored during the course of infection (mean ± SEM of 6–9 mice per group). (G) Lung virus titer at day 5 p.i.

AM were described to maintain independently from blood monocytes through self-renewal [31]. We and others recently showed that AM originate from a fetal monocyte precursor in the lung around E17–19, which subsequently differentiates into mature AM during the following 5–7 days ([32]; Schneider et al., unpublished data). Single intranasal transfer of CD45^+^ lung cells from E18.5 fetuses into *Csf2rb* ^−/−^ newborns completely restored development of mature AM (Figure 5A, B) but did not contribute to the pool of BM-derived short-lived DCs including CD103^+^ DCs 6 weeks after transfer (Figure 5C). Thus, we generated mice lacking *Csf2rb* in every cell but AM. Presence of wild-type AM in *Csf2rb* ^−/−^ mice protected from pulmonary proteinosis, severe morbidity and mortality following influenza infection (Figure 5 D–F). Moreover, viral load in *Csf2rb* ^−/−^ mice with restored AM compartment was reduced to levels observed in wild type controls (Figure 5G). These results unequivocally demonstrate that the absence of AM is responsible for disease severity and rule out a putative contribution of DCs or other immune cells in mice lacking GM-CSF or its receptor.

### Selective AM depletion results in respiratory failure and increased morbidity following influenza virus infection

**Figure 6:**
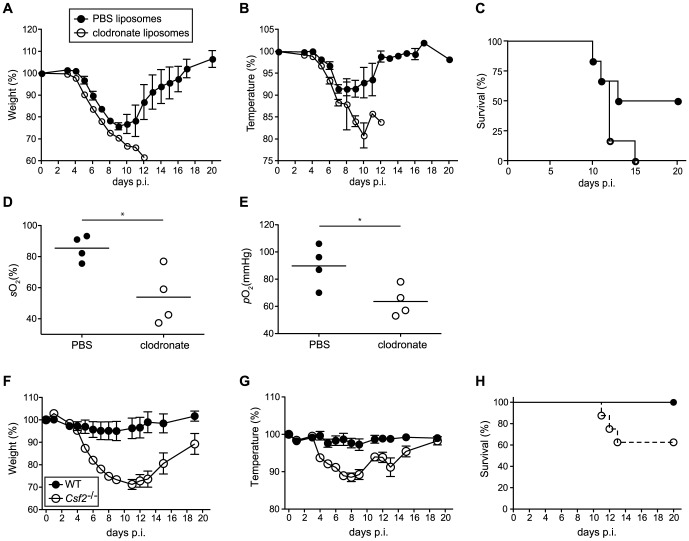
Increased morbidity and respiratory failure in mice upon selective depletion of alveolar macrophages prior to pulmonary virus infection. (A–E) Mice were treated with clodronate or control PBS liposomes 2 days prior to infection with 50 pfu PR8 influenza virus. Loss of body weight (A) and temperature (B) was monitored. (C) Survival was assessed over a period of 3 weeks after infection. Values indicate mean ± SEM of 6 mice per group. Arterial blood oxygen saturation (D) and O_2_ partial pressure (E) was measured in infected animals at d8. (F–H) WT and *Csf2* ^−/−^ mice were infected with 10^4^ pfu vaccinia virus WR i.t. Loss of body weight (F), loss of temperature (G) and survival (H) was monitored. Values indicate mean ± SEM of 7–8 mice per group.

To further rule out that a defect in CD103^+^ DCs contributed to the susceptibility of *Csf2* ^−/−^ mice to influenza virus infection, we selectively depleted AM in wild-type mice prior to infection using clodronate liposomes. Although DC subsets remained unaffected in clodronate-treated mice (Figure S5), AM-depletion resulted in increased morbidity (i.e. temperature and weight loss) (Figure 6A, B) and mortality (Figure 6C), which was associated with hypoxia (Figure 6D, E) basically resembling the phenotype observed in *Csf2* ^−/−^ mice. These results further support the conclusion that absence of AM and respiratory failure rather than impaired CD103^+^ DC-mediated CD8^+^ T cell responses are responsible for the high morbidity of *Csf2* ^−/−^ mice to influenza virus infection.

To assess whether the AM play an important role in resistance to other pulmonary viral infections, we assessed the outcome of infection with vaccinia virus (WR) in *Csf2* ^−/−^ and control WT mice. Control of vaccinia virus mainly depends on innate and T helper cell mediated B cell antibody responses, whereas the cytotoxic T cell response plays only a marginal role [33], [34]. When infected with a virus dose that did not induce any morbidity in WT mice, *Csf2* ^−/−^ mice showed a pronounced loss of body weight and temperature (Figure 6F, G) starting already during the innate immune response at day 4 p.i. and displayed increased lethality (Figure 6H), reminiscent to the outcome of influenza virus infection. Altogether, these results establish a direct role of AM in prevention of fatal respiratory viral infections.

### Mice with immature and dysfunctional alveolar macrophages due to the absence of PPARγ show increased mortality and reduced lung function following influenza infection

**Figure 7:**
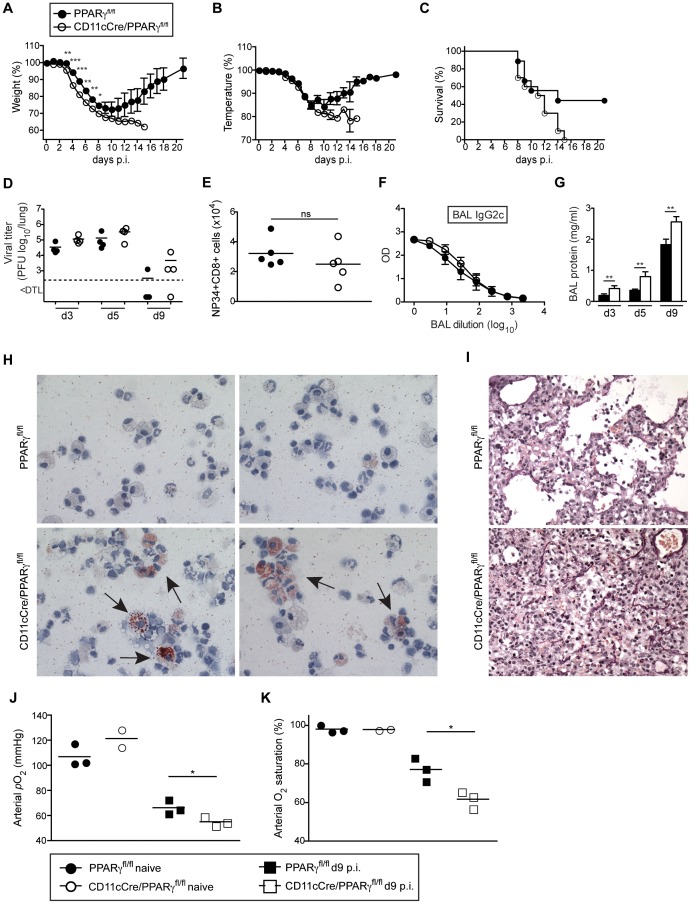
CD11c-Cre/*Pparg^fl/fl^* mice have a reduced resistance to influenza virus infection despite an intact antiviral adaptive response. *Pparg* ^fl/fl^ and CD11c-Cre/*Pparg* ^fl/fl^ mice were infected i.t. with 250 pfu PR8 influenza virus. Loss of body weight (A), temperature (B) and survival (C) was monitored. Values indicate mean ± SEM of 9–10 mice per group. (D–K) For the characterization of the anti-viral immune response, *Pparg* ^fl/fl^ and CD11cCre/*Pparg* ^fl/fl^ mice were infected i.t. with 50 pfu PR8 influenza virus. Symbols represent values of individual mice and the mean is indicated. (D) The virus titers in the lung were determined at d3, d5 and d9 after infection. (E) Total numbers of influenza NP34-specific CD8^+^ T cells in the BAL were analyzed at d10 post-infection. (F) Influenza HA-specific IgG2c antibody concentrations in the BAL at d13 post-infection were determined by ELISA. Values indicate mean ± SEM of 4–5 mice per group. (G) Total protein in the BAL was measured at indicated time points. (H) Cytospins of BAL isolated from infected mice at d9 were stained with Oil Red O. Micrographs were taken at 63× magnification. Representative pictures of 2 individual mice are shown. (I) Panels show lung sections of day 9-infected mice stained using the Verhoeff-Van Gieson protocol. (J and K) Lung function was measured in naïve and day 9-infected animals. Arterial blood oxygen partial pressure (J) and O_2_ saturation (K) are shown.

Mice lacking *Pparg* specifically in macrophages (LysM-Cre/*Pparg* ^fl/fl^) develop pulmonary alveolar proteinosis [35]. Moreover, using CD11c-Cre/*Pparg* ^fl/fl^ mice that lack *Pparg* in AM and DCs, we recently found that development of AM is arrested in an immature state with a defect in surfactant catabolism and lipid metabolism while development of DC subsets remained unaffected (data not shown). To determine whether absence of functional AM affects resistance of CD11c-Cre/*Pparg* ^fl/fl^ mice to respiratory viral infection, we infected WT and CD11c-Cre/*Pparg* ^fl/fl^ mice with influenza virus PR8. Compared to WT, CD11c-Cre/*Pparg* ^fl/fl^ mice showed an earlier onset and more pronounced loss of body weight and body temperature (Figure 7A, B) associated with increased lethality (Figure 7C), although lung virus titers were insignificantly elevated (Figure 7D). Besides a slightly increased number of neutrophils, recruitment of inflammatory cells to the lung was in general normal in CD11c-Cre/*Pparg* ^fl/fl^ mice (Figure S6). Moreover, we found similar frequencies and total numbers of anti-viral CD8^+^ T cells as well as IFNγ-producing CD4^+^ and CD8^+^ T cells (Figure 7E and Figure S6) indicating that DC-mediated T cell priming and effector responses were unaffected in CD11c-Cre/*Pparg* ^fl/fl^ mice. Likewise, levels of anti-influenza HA-specific antibody levels (i.e. IgG2c, IgA and IgG1) were comparable in BAL and serum of CD11c-Cre/*Pparg* ^fl/fl^ and WT mice (Figure 7F and Figure S6). Together these results indicate that innate and adaptive anti-viral immune responses were largely intact in CD11c-Cre/*Pparg* ^fl/fl^ mice.

Lungs of infected CD11c-Cre/*Pparg* ^fl/fl^ mice contained increased amounts of total protein in BAL fluid with a high proportion of lipid-engorged foam cells and alveoli filled with cellular infiltrate and debris (Figure 7G–I and Figure S6). Moreover, CD11c-Cre/*Pparg* ^fl/fl^ mice displayed significantly lower lung function as measured by arterial oxygen levels (Figure 7J, K). Taken together, CD11c-Cre/*Pparg* ^fl/fl^ mice with immature and dysfunctional AM show increased morbidity and respiratory failure due to an impaired removal of dead cells and debris following influenza virus infection similar to, but not as pronounced as *Csf2* ^−/−^ mice that are completely devoid of AM.

### Influenza virus infection potently upregulates expression of interferon-induced transmembrane protein 3 in alveolar macrophages

**Figure 8:**
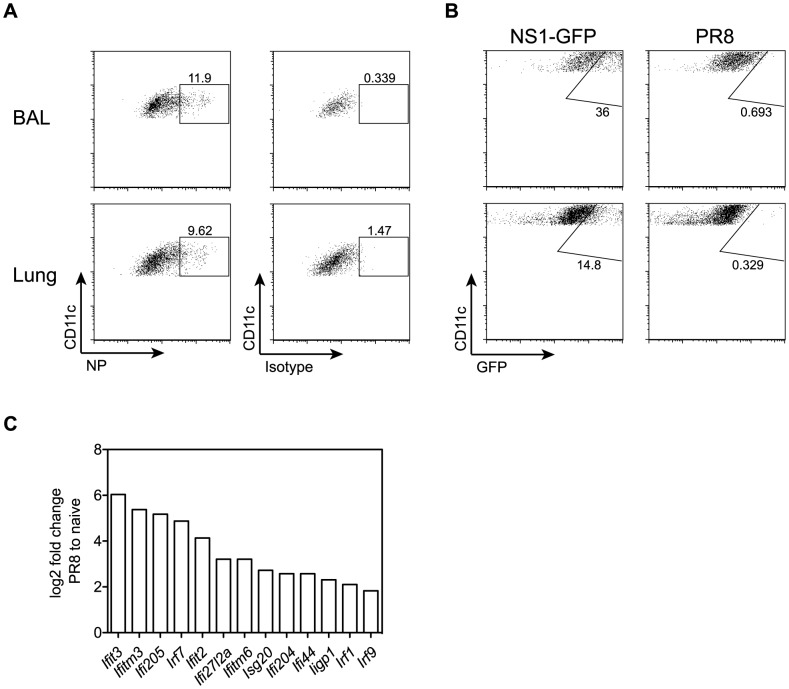
Influenza infection potently induces expression of interferon-regulated antiviral factors in AM. (A) Mice were infected with 50 pfu PR8 influenza virus. Intracellular NP expression was measured by flow cytometry in CD11c^+^autofluorescent AM isolated from BAL and lung 5 days after infection. (B) Mice were infected with 10^6^ pfu NS1-GFP virus [51] or 10^3^ pfu PR8. GFP expression was analyzed in AM isolated from BAL and lung 5 days after infection. (C) Microarray analysis of sorted AM from lungs of naive or influenza-infected animals at d5 post-infection with 50 pfu PR8. Bar graphs show relative expression levels of various interferon-induced genes plotted as log_2_-fold change in AM from infected lungs compared to naïve. The mean of two microarray samples per condition is shown. For each sample, AM from two individual mice were pooled. Differences in expression levels were validated by qPCR for most of the depicted genes (i.e. *Ifitm3*, *Ifitm6*, *Ifit2*, *Ifit3* and *Ifi205*).

GM-CSF-deficient mice lacking alveolar macrophages showed 5–10-fold increased viral titers and a delayed viral clearance. Therefore, we next asked whether alveolar macrophages might contribute directly to viral clearance besides their role in prevention of respiratory failure following viral infection. Infection of C57BL/6 mice with PR8 or NS1-GFP influenza virus showed a considerable proportion of AM containing viral material as determined by staining of viral NP or expression of NS1-GFP (Figure 8A, B). In addition, comparison of gene expression profiles of sorted AM from naïve and influenza virus infected mice by microarray analysis showed that the latter displayed a typical interferon signature with a 4- to 65-fold upregulation of several interferon-induced genes including the interferon-induced transmembrane protein 3 (*Ifitm3*) and *Ifitm6*, which were upregulated 42- and 9-fold, respectively (Figure 8C). *Ifitm3* has recently been described as a key component in restricting viral spread and the morbidity and mortality following influenza virus infection [36]. Thus it is tempting to speculate that AM act like a virus sink and prevent morbidity at least partially through Ifitm3.

## Discussion

In this study, we revisited the role of GM-CSF in AM homeostasis and function of this cell population in respiratory viral infection. According to the current understanding, *Csf2*- and *Csf2rb*-deficient mice develop pulmonary alveolar proteinosis (PAP) due to a defect in terminal maturation of AM involving impaired lipid catabolism. However, using 8-parameter flow cytometry in combination with dead cell exclusion, we found that *Csf2* ^−/−^ mice were completely devoid of AM and presented a massive accumulation of dead CD45-negative cells, which are presumably epithelial cells, in the BAL and lung consistent with an important role of AM in removal of dead cells (efferocytosis). Indeed, usage of a viability dye in combination with CD45 staining was inevitable for the exclusion of highly auto-fluorescent dead cells and avoidance of misidentification as AM. We also observed increased numbers of neutrophils in the BAL of naive *Csf2* ^−/−^ mice supporting an association of impaired efferocytosis and chronic inflammatory lung disease [37]. Using mixed bone marrow chimeras we demonstrated that AM exclusively differentiated from WT and not from *Csf2rb* ^−/−^ BM. The absence of *Csf2rb* ^−/−^ AM in mixed BM chimeras also excludes the possibility that these cells have developed and died subsequently due to a functional defect in surfactant metabolism and accumulation of cellular debris, as the mixed chimeras did not develop PAP pathology. These results demonstrate a cell intrinsic requirement of GM-CSFR signaling for licensing development of AM from a precursor cell. Our results are in line with a recent report describing highly reduced numbers of AM in *Csf2* ^−/−^ mice and a requirement of GM-CSF signaling for the reconstitution of AM post-irradiation [28]. The license is provided by GM-CSF secretion of radio-resistant lung cells, as shown by our 4-way BM chimeras. Whether during homeostasis GM-CSF also influences the functionality of AM needs to be determined using inducible knock out strategies or antibody-mediated neutralization.

The role of GM-CSF for the development of DCs has been extensively studied. Most lymphoid tissue DCs develop normally in the absence of GM-CSF *in vivo* [38]. In contrast, GM-CSF has been shown to play a pivotal role for the development of non-lymphoid tissue DCs in the lamina propria [39] and the skin [40]. A recent report suggested that GM-CSF is critical for the homeostasis of tissue-resident CD103^+^ DCs in particular in the lung and skin [25], while others found that GM-CSF merely regulates CD103 expression levels on DCs and not development of this DC subset [26]. In keeping with the latter study by Edelson *et al.*, we found that total CD11b^−^CD103^+^ DC cell numbers were not affected in the lung of adult *Csf2*-deficient mice, while CD103 surface expression on CD11b^−^ DCs was clearly reduced. Interestingly, in lungs of neonatal *Csf2* ^−/−^ mice the CD103^+^ DC subpopulation was strongly reduced in cell numbers. Similarly, the vast majority of CD103^+^ DCs present in lungs of mixed bone marrow chimeras originated from WT but not *Csf2rb* ^−/−^ bone marrow and the few *Csf2rb* ^−/−^ BM-derived CD11b^−^ lung DCs were CD103^low^. Whether impaired CD103 expression affects DC function is not clear. CD103^+^ DCs possess a unique potential in phagocytosis of virus-infected epithelial cells and capacity in cross-presentation of viral antigens to CD8^+^ T cells [41]. Several reports suggested that CD103^+^ DCs are the main inducers of CD8^+^ T cell responses to influenza virus infection with supportive evidence mainly based on depletion experiment using langerin-DTR mice [19], [30], [42]. It should be noted, however, that langerin is also expressed on CD8α^+^ DCs from spleen and LNs [43], [44], which are accordingly also depleted in langerin-DTR mice [45]. Indeed, LN-resident CD8α^+^ DCs as well as CD11b^+^ lung tissue DCs have also been implicated in influenza antigen transport, presentation and CD8^+^ T cell priming [30], [46], [47]. *Batf3*-deficient mice that are devoid of both CD8α^+^ DCs and CD103^+^ DCs showed a substantial reduction in anti-viral CD8^+^ T cell responses indicating that both CD8α^+^ DCs in spleen and lymph node together with lung migrating CD103^+^ DCs contribute to the induction and maintenance of T cell responses to influenza virus. In contrast, despite an almost 2-fold reduction in frequencies, total lung CD103^+^ DC numbers in influenza virus-infected *Csf2* ^−/−^ mice were comparable to wild-type mice and accordingly, antiviral CD8^+^ T cell numbers and cytokine responses in the BAL, lung, and the draining LN remained unaffected. However, the influenza-induced pathology and morbidity was far worse in *Csf2*-deficient mice lacking AM but having intact B and T cell responses as compared to *Batf3*-deficient mice with constrained CD8^+^ T cell responses due to the absence of both CD103^+^ DCs and CD8α^+^ DCs but intact AM indicating that only the latter are critical for survival of respiratory viral infection.

PAP was strikingly aggravated in influenza virus infected compared to naive *Csf2* ^−/−^ mice. The accumulation of cellular debris, dead epithelial cells and surfactant material in the respiratory tract in the absence of AM resulted in impaired gas exchange and eventually in a fatal hypoxia. Similar results were obtained by clodronate-mediated depletion of macrophages without affecting DCs in wild-type mice before viral infection. In keeping with our data, transgenic mice with lung-restricted GM-CSF overexpression (SPC-GM) and increased AM numbers [48] were better protected from influenza virus-induced morbidity [24]. However, using the same transgenic mice, another study linked the GM-CSF-induced protection to enhanced CD103^+^ DC-mediated antiviral T cell responses, although numbers of DCs and virus-specific CD4^+^ and CD8^+^ T cells in SPC-GM mice were comparable to non-transgenic WT mice and the viral load was significantly reduced as early as day 3 post-infection prior CD8^+^ T cell expansion and effector function [27]. Moreover, this study concluded that increased susceptibility of *Csf2* ^−/−^ mice is a consequence of defective CD103^+^ DC-mediated CD8^+^ T cell responses [27]. In contrast, protection from virus-induced pathology and mortality by reconstitution of AM development in *Csf2rb* ^−/−^ mice (Figure 5) unequivocally demonstrates that the absence of AM is the underlying reason for hypersusceptibility to influenza virus in mice lacking GM-CSF or its receptor. Notably, *Csf2* ^−/−^ mice showed also increased morbidity and pronounced lethality in response to pulmonary vaccinia virus infection, which does not depend on CD8^+^ T cells [33], [34]. These data suggest a vital role of AM in resistance to respiratory viral infection in general, although it should be noted that depletion of AM in mice infected with RSV did not alter subsequent disease development[49].

Induction of host cell death is a hallmark of viral infection including influenza virus infection. Indeed, increased cell death has been associated with the pathogenicity of highly virulent influenza virus [50]. Moreover, we found a correlation between viral burden, PAP, and severity of hypoxia in wild-type mice. Our findings suggest that the pivotal function of AM is the removal of dead cells and cellular debris to prevent clogging of the airways and maintain gas exchange during respiratory viral infection. In addition, AM may contribute to viral clearance or interfere with virus-induced morbidity by yet unknown mechanisms. Consistent with previous reports [51] [19], we found that influenza virus can directly infect AM (Figure 8A,B). Interestingly, transcriptome analysis of sorted AM from infected and uninfected mice revealed a striking upregulation of interferon response signature genes including high levels of *Ifitm3*. Ifitm3 has recently been described as a key component in restricting viral spread and the morbidity and mortality following influenza virus infection [36]. Moreover, high *Ifitm3* expression in influenza-specific lung-resident CD8^+^ memory T cells confers resistance to infection and enhances survival of these cells upon recall infection with the virus [52]. Thus, induction of *Ifitm3* in AM could serve as a mechanism to promote AM survival and thereby limit the loss of this vital cell type during influenza infection. Furthermore and in addition to their crucial role in maintaining respiratory function, AM could have a direct antiviral role serving as a sink for influenza virus consistent with slightly elevated virus titers in mice lacking AM.

Taken together, we identified a key function of alveolar macrophages in phagocytosis of dead cells and maintenance lung function in respiratory viral infections. Mice lacking *Csf2* or *Csf2rb* are highly vulnerable to influenza virus infection due to the absence of AM but not potentially impaired DC/T cell immunity. These results have implications for therapies targeting Csf2 (GM-CSF).

## Materials and Methods

### Mice

*Csf2* ^−/−^ mice (originally kindly provided by A. Dunn, Ludwig Institute for Cancer Research, Royal Melbourne Hospital, Victoria, Australia) [7] were backcrossed to BALB/c for 7 generations [53]. *Csf2* ^−/−^ (C57BL/6) and *Csf2rb* ^−/−^ (C57BL/6) mice were kindly provided by B. Becher (University Zurich). C57BL/6 and BALB/c mice were purchased from Charles River (Germany). It should be noted that most of the results shown were done with *Csf2* ^−/−^ (C57BL/6) and validated with *Csf2* ^−/−^ (BALB/c). B6.129S(C)-*Batf3^tm1Kmm^*/J (*Batf3* ^−/−^) mice [54] were purchased from the Jackson Laboratory. *Pparg* ^fl/fl^ mice [55] originally kindly provided by P. Chambon (Université Louis Pasteur, Illkirch Cedex, France) and backcrossed for 6 generations to C57BL/6 before crossing to CD11c-Cre [56] mice in our facility to generate CD11c-Cre/*Pparg* ^fl/fl^ mice. All animals were housed in individually ventilated cages under specific pathogen free conditions at BioSupport AG (Zurich, Switzerland) and used for experiments at between 8 and 12 weeks of age.

#### Ethics statement

All animal experiments were approved by the local animal ethics committee (Kantonales Veterinärsamt Zürich, licenses 167/2011 and 113/2012), and performed according to local guidelines (TschV, Zurich) and the Swiss animal protection law (TschG).

### Cells suspension preparations

Mice were sacrificed by an overdose of pentobarbital sodium i.p. BAL was isolated by canalization of the trachea with a catheter. The lungs were flushed with 3×400 µl PBS and BAL cells were harvested by centrifugation. Lungs were digested with 2 mg/ml of type IV collagenase (Worthington) and 0.02 mg/ml DNaseI (Sigma) at 37°C for 45 minutes and subsequently passed through a 70 µm cell strainer.

### Flow cytometry

Multiparameter analysis was performed on a FACSCanto II or LSR Fortessa (BD) and analyzed with FlowJo software (Tree Star). Monoclonal antibodies specific to mouse CD11c (N418), CD11b (M1/70), Ly-6C (HK1.4), Siglec-F (E50-2440, BD Biosciences), CD103 (2E7), CD115 (AFS98, eBioscience), CD45 (30-F11), CD45.1 (A20), CD45.2 (104), CD4 (GK1.5), CD8α (53-6.7), MHC class II (M5/114.15.2, eBioscience), Gr-1 (RB6-8C5, eBioscience), CD49b (DX5, eBioscience), CD69 (H1.2F3), TNF-α (MP6-XT22), IFN-γ (XMG1.1) were purchased from Biolegend unless otherwise stated. Dead cells were stained using eFluor780 (eBioscience). PE-conjugated peptide-MHC class I tetramers (H-2Db/NP34) with the peptide NP34 (NP366-374; ASNENMETM) from the nucleoprotein of influenza virus A/PR/8/34 were generated as described [57]. For detection of intracellular NP expression, cells were incubated with a monoclonal mouse anti-influenza NP antibody (HB-65, homemade) followed by staining with AF647-conjugated anti-mouse IgG (Life Technologies Co.). Prior to all stainings, FcγIII/II receptors were blocked by incubation with homemade anti-CD16/32 (2.4G2).

### Phagocytosis of apoptotic cells

Thymocytes were isolated from C57BL/6 mice and apoptosis was induced by exposure to 60 mJ UV radiation (Spectrolinker XL-1500; Spectronics Corporation). After 2 h incubation at 37°C in IMDM+10% FCS, cells were labeled with 5 µM eFluor670 (eBioscience) according to the manufacturer's instructions, washed extensively with IMDM+10% FCS and delivered i.t. in PBS. 3 and 24 h after administration, efferocytosis by cells in the BAL and lung was assessed by flow cytometry.

### Bone marrow chimeras

For mixed bone marrow chimeras, CD45.1^+^CD45.2^+^ mice were lethally irradiated (9.5 Gy, using a caesium source) and reconstituted with 5–10×10^6^ BM cells of a 1∶1 mixture of CD45.1^+^WT∶CD45.2^+^WT or CD45.1^+^WT∶CD45.2^+^ *Csf2rb* ^−/−^. Mice were analysed 10 weeks post-reconstitution. For *4-way Csf2* ^−/−^/WT bone marrow chimeras, WT and *Csf2* ^−/−^ mice were lethally irradiated and reconstituted with 5–10×10^6^ *Csf2* ^−/−^ or WT BM cells. Mice were analysed 16 weeks post-reconstitution.

### BAL protein and fatty acid analysis

Total BAL protein concentration was measured by BCA Protein Assay (Thermo Scientific) according to the manufacturer's instructions. For fatty acid analysis, BAL lipids were extracted as described by Moser *et. al* [58] using methanol-methylene chloride (3∶1, v/v). Thereafter, two internal standards, deuterated C17:0 (Cambridge Isotope Laboratories, Inc., Andover, MA, USA) and deuterated C22:0 (Dr. Ehrenstorfer GmbH, Augsburg, Germany) were added to the samples. Derivatization was done by adding acetyl chloride followed by an incubation at 100°C for 1 h. The reaction was stopped with 7% K_2_CO_3_ and the extraction of fatty acid methyl esters was performed with hexane. After centrifugation for 20 min at 2500 rpm, the hexane layer was dried under a continuous nitrogen stream and resuspended in heptane. For the fatty acid analysis, gas chromatography-mass spectrometry was applied using a Finnigan PolarisQ ion trap gas chromatography-mass spectrometry system (Thermo Quest, Austin, TX, USA). For quantification of fatty acid methyl esters, the specific masses were extracted. Analytes were identified with authentic standards by comparison of their retention time and their mass spectrum.

### Viruses and infection

Influenza virus strain PR8 (A/Puerto Rico/34, H1N1) was originally provided by J. Pavlovic, University Zurich. The influenza strain PR8 NS1-GFP carrying a GFP reporter in the NS segment was kindly provided by A. García-Sastre [51]. Vaccinia virus WR was kindly provided by J. Mercer, ETH Zurich. For infections, the mice were anaesthetized and intratracheally inoculated with indicated doses of virus in 50 µl endotoxin-free PBS. To determine influenza virus titers in the lungs, samples were collected on various days after infection, homogenized and serially diluted with MDCK cells as described [59]. Infected cells were detected using a monoclonal mouse anti-influenza NP antibody (HB-65, homemade). For the depletion of AM, mice were treated with 100 µl clodronate liposomes i.t. 2 days prior to infection. Clodronate was a gift from Roche Diagnostics GmbH and liposomes were prepared as previously described [60]. Control mice were treated with PBS liposomes.

### *In vitro* stimulation

For restimulation, 1.5×10^5^ bone marrow-derived dendritic cells (BMDC) were incubated overnight with 1×10^6^ pfu UV-inactivated PR8 virus in 96-well plates. BMDC were pulsed with 1 µg/mL NP147 (Balb/c) or NP34 (C57BL/6) peptide for 2 hours before BAL, lung or LN cells from individual mice were added and restimulation was performed for 4–5 h in the presence of 2 µM monensin (Sigma-Aldrich). After surface staining and formalin-fixation, intracellular cytokine staining was done in the presence of 0.5% saponin using anti-mouse TNF-α FITC and IFN-γ APC and analysed by flow cytometry.

### Detection of virus-specific antibodies

Serum or BAL fluid from indicated time points post-infection was measured for influenza HA-specific antibody levels. Ninety-six well plates (Maxisorp; Nunc) were coated with 5 µg/ml recombinant PR8 influenza virus HA (a kind gift of M. Bachmann, Cytos) in PBS overnight at 4°C. After blocking, serum and BAL fluid from individual mice were serially diluted and incubated at RT for 2 hours. Plates were washed and incubated with alkaline-phosphatase-labelled goat anti-mouse isotype-specific antibodies (Southern Biotech Technologies, Inc.) and developed using substrate p-nitrophenyl phosphate (Sigma-Aldrich). Optical densities were measured on an enzyme-linked immunosorbent assay reader (Bucher Biotec) at 405 nm.

### Measurement of arterial oxygen saturation

The femoral artery was catheterized in anaesthetized (2% isoflurane in oxygen) mice and the wound was locally anaesthetized by the application of 2% lidocaine before the cut was closed and the catheter was sewn to the thigh to be held in place. The application of isoflurane was stopped and mice regained consciousness and were kept restrained in a dark card tube while normally breathing room air for 10 min to equilibrate blood gas. Subsequently, 100 µL arterial blood was taken from the catheter and blood gas composition was measured on an ABL800Flex blood gas analyzer (Radiometer, Denmark) before mice were sacrificed.

### Lung histology

The lungs were removed, fixed in formalin and processed for Hematoxylin and Eosin (H&E) staining. Histological sections were evaluated according to general inflammation.

### Cell sorting and transfer

Fetal CD45^+^ cells were sorted from the lungs of CD45.1^+^ wild-type E18.5 fetuses using a FACSAria IIIu (BD). Neonatal *Csf2rb* ^−/−^ recipient mice were anesthetized using Isoflurane and 1×10^5^ fetal cells were administered i.n. in 10 µl PBS. Reconstitution of AM in the BAL and lung was assessed by flow cytometry 6 weeks post-transfer and mice were used for infection experiments at 8 weeks of age.

### Microarray analysis

Lungs of naive or influenza-infected animals at d5 post-infection were processed as described and stained with eF780, anti-mouse CD45, CD11c, CD11b and Siglec-F. AM were sorted as eF780^−^CD45^+^CD11c^high^autofluorescence^high^Siglec-F^+^ (BD FACSAria IIIu) and RNA was prepared using PureLink RNA Mini Kit (ambion, Life Technologies Co.), amplified and hybridized on the Affymetrix Mouse Gene 1.1 ST.

### Statistical analysis

Mean values, SD, SEM, and Student's t test (unpaired) were calculated with Prism (GraphPad Software, Inc). p<0.05 (*), p<0.01 (**), p<0.001 (***), p<0.0001 (****).

## Supporting Information

Figure S1 **Alveolar macrophages are the predominant efferocytic cell population in the bronchoalveolar space and are completely absent in** ***Csf2*** **−/− mice.** (A) Dot plots depict identification of AM as CD11c^+^Siglec-F^+^ cells with high auto-fluorescence and assessment of viability using the viability dye eFluor780 (that labels dead cells) in BAL and lung of *Csf2* ^−/−^ and WT mice. Note that the few remaining CD11c^+^Siglec-F^+^ cells in *Csf2*−/− mice are all dead hematopoietic cells with high fluorescence. A maximal amount of 20'000 events per plot is depicted. (B and C) C57BL/6 mice were inoculated i.t. with eFluor670-labeled apoptotic thymocytes. BAL and lung cells were isolated 3 and 24 h later and analyzed by flow cytometry. (B) Dot plots show the frequencies of AM among all eFluor670^+^ efferocytic cells in the lung. (C) Percentages of eFluor670^+^ cells among AM in the lung. Representative samples are shown (n = 4).
(EPS)

Figure S2 **GM-CSF produced by radio-resistant cells drives development of alveolar macrophages.** (A–C) Pulmonary alveolar proteinosis in WT→WT, WT→*Csf2* ^−/−^, *Csf2* ^−/−^→WT and *Csf2* ^−/−^→*Csf2* ^−/−^ BM chimeras was assessed by measurement of BAL total protein (B), palmitic acid (C) and cholesterol concentration (D). Bar graphs represent the mean ± SD (WT→WT, n = 4; WT→*Csf2* ^−/−^, n = 3; *Csf2* ^−/−^→WT, n = 3; *Csf2* ^−/−^→*Csf2* ^−/−^, n = 1). (D) Mixed BM chimeras (described in Figure 1 J and K) were analyzed for the contribution of CD45.1 and CD45.2 BM to the development of lung CD11c^−^CD11b^+^ and blood myeloid subsets. The mean ± SD is shown (n = 3).
(EPS)

Figure S3 **GM-CSF produced by radio-resistant cells is crucial for resistance to influenza infection.** (A and B) WT→WT, WT→*Csf2* ^−/−^, *Csf2* ^−/−^→WT and *Csf2* ^−/−^→*Csf2* ^−/−^ BM chimeric mice were infected i.t. with 50 pfu PR8 influenza virus. Loss of body temperature (A) and survival (B) was monitored. Values indicate mean ± SEM (WT→WT, n = 3; WT→*Csf2* ^−/−^, n = 6; *Csf2* ^−/−^→WT, n = 5; *Csf2* ^−/−^→*Csf2* ^−/−^, n = 6).
(EPS)

Figure S4 **Intact anti-viral B and NK cell responses in** ***Csf2*** **^−/−^ mice.** WT, *Csf2* ^−/−^ and *Batf3* ^−/−^ mice were infected i.t. with 50 pfu PR8 influenza virus. (A) The virus titers in the lung at d5 and d13 after infection were measured by plaque-assay. (B) Influenza HA-specific antibody levels of the indicated Ig isotypes in the BAL and serum of WT and *Csf2* ^−/−^ mice at d10 after infection were determined by ELISA. (C and D) Bar graphs display total numbers of DX5^+^ NK cells (C) and frequencies of CD69^+^DX5^+^ cells (D) at day 3 and 5 post-infection. Values indicate mean ± SD of 3–5 mice per group.
(EPS)

Figure S5 **Clodronate treatment selectively depletes AM.** (A–E) Lungs of mice treated with clodronate liposomes were analysed for AM and CD103^+^ DCs 2 days after treatment. (A) Plots show frequencies of AM in the BAL and lung gated on eF780^−^CD45^+^ cells. Bar graphs display total cell numbers of AM in BAL (B) and lung (C). (D and E) Frequencies (D) and total cell numbers (E) of CD103^+^ and CD11b^+^ DCs among CD11c^+^Siglec-F^−^ cells in the lung are shown. Bar graphs represent the mean ± SD (n = 4).
(EPS)

Figure S6 **Lung inflammatory infiltrate and adaptive immune responses in CD11cCre/Pparg^fl/fl^ mice infected with influenza virus.** CD11cCre/*Pparg* ^fl/fl^ and control *Pparg* ^fl/fl^ mice were infected i.t. with 50 pfu PR8 influenza virus. (A) Differential cell composition in the BAL was assessed by flow cytometry at d6 post-infection. (B–D) Anti-viral T cell response was analyzed at d10 post-infection. Total numbers of influenza NP34-specific CD8^+^ T cells in the lung-draining lymph node (B) and frequencies in the BAL (C) were measured (mean ± SD, n = 5). (D) IFN-γ production in BAL CD8^+^ and CD4^+^ T cells was analyzed by virus-specific restimulation. (E) Influenza HA-specific antibody concentrations of the indicated isotypes in the BAL and serum were determined by ELISA. (F) Percentages of oil red O^+^ cells quantified cytospins of the BAL at d9 after infection. Symbols represent values of individual mice and the mean is indicated. Bar graphs show the mean ± SD of 5 mice per group.
(EPS)
